# *MicroRNA* Variants Increase the Risk of HPV-Associated Squamous Cell Carcinoma of the Oropharynx in Never Smokers

**DOI:** 10.1371/journal.pone.0056622

**Published:** 2013-02-15

**Authors:** Xicheng Song, Erich M. Sturgis, Jun Liu, Lei Jin, Zhongqiu Wang, Caiyun Zhang, Qingyi Wei, Guojun Li

**Affiliations:** 1 Department of Head and Neck Surgery, The University of Texas MD Anderson Cancer Center, Houston, Texas, United States of America; 2 Department of Otorhinolaryngology and Head and Neck Surgery, Yuhuangding Hospital of Qingdao University, Yantai, China; 3 Department of Epidemiology, The University of Texas MD Anderson Cancer Center, Houston, Texas, United States of America; 4 Department of Otolaryngology Head and Neck Surgery, West China Hospital, Sichuan University, Chengdu, China; 5 Department of Stomatology, Jinling Hospital, School of Medicine, Southern Medical University, Nanjing, China; 6 Department of Radiology, East Hospital, Tongji University School of Medicine, Shanghai, China; 7 Department of Otorhinolaryngology Head and Neck Surgery, Changhai Hospital, The Second Military Medical University, Shanghai, China; Hannover Medical School, Germany

## Abstract

**Background:**

Both *microRNAs* and human papillomavirus (HPV) infection play an important role in the development and progression of oral squamous cell carcinoma (OSCC). In addition, *microRNAs* affect all facets of the immune/inflammation responses to infection, which may control HPV clearance. We thus hypothesized that *microRNA* polymorphisms modify the association between HPV16 seropositivity and OSCC risk.

**Methods:**

Four single-nucleotide polymorphisms in *microRNAs* were genotyped and HPV16 serology was determined in 325 cases and 335 matched controls. Odds ratios (ORs) and 95% confidence intervals (CIs) were calculated using univariate and multivariable logistic regression models.

**Results:**

Overall, each polymorphism had no significant main effect on OSCC risk. Compared with the risk among individuals with both *miR146* rs2910164 GG genotype and HPV16 seronegativity, risk of OSCC was increased among those with CG or CC genotype and HPV16 seronegativity (OR, 1.2; 95% CI, 0.9–1.8), GG genotype and HPV16 seropositivity (OR, 3.0; 95% CI, 1.8–5.0), and CG or CC genotype and HPV16 seropositivity (OR, 4.7; 95% CI, 2.3–9.4). Similar results were found for *miR149* rs2292832, *miR196* rs11614913, and *miR499* rs3746444. Analyses stratified by tumor sites and smoking status showed that each polymorphism significantly increased the risk of HPV16-associated squamous cell carcinoma of the oropharynx (SCCOP), and such effect modification was particularly prominent in never smokers.

**Conclusions:**

Our results indicate that *microRNA* polymorphisms modify the risk of OSCC associated with HPV16 seropositivity, particularly in patients with SCCOP and never smokers. Larger studies are needed to verify our findings.

## Introduction

Oral squamous cell carcinoma (OSCC; SCC of the oropharynx, SCCOP and SCC of oral cavity) accounts for the majority of head and neck malignant tumors and is one of the most common malignancies worldwide. It has been estimated that approximately 36,540 new cases of OSCC will be diagnosed and 7,880 individuals will die from these cancers in 2012 in the United States [1]–[3]. Tobacco use and alcohol consumption are considered the most important risk factors for OSCC [4]. Despite decreasing smoking and drinking rates in the United States, the overall incidence of OSCC in young adults has been increasing in recent decades. Epidemiologic evidence implies that this increase is related to an increasing prevalence of infection with human papillomavirus (HPV), partly due to change in sexual behaviors [5]–[7]. Therefore, HPV may be another important etiologic factor for OSCC in addition to tobacco and alcohol use. Of the more than 130 types of HPV, the high-risk HPV type 16 (HPV16) is by far the most commonly associated with head and neck cancers, accounting for about 90% of HPV-associated OSCCs [8]–[10]. Although HPV infection may be a major risk factor for OSCC, only a small fraction of individuals infected with HPV eventually develop the malignancy, implying that host genetic factors may modify the association between HPV infection and OSCC risk.

*MicroRNAs* are highly conserved, single-stranded, short, noncoding RNAs of about 22 nucleotides that regulate gene expression through completely or partially base pairing with target mRNAs at the 3′-untranslated region, resulting in mRNA cleavage or translational suppression [11]. To date, emerging evidence has demonstrated that *microRNAs* play key roles in a broad range of physiologic and pathologic processes [12], [13]. Although the biologic functions of *microRNAs* remain largely unclear, previous studies have indicated that *microRNAs* may participate in tumorigenesis, functioning as tumor suppressors and/or oncogenes [14]–[18], and affect the etiology, diagnosis, and prognosis of various cancers [17], [19]–[25]. Furthermore, *microRNAs* may be involved in regulation of the immune and inflammatory response systems [26], [27], which may control HPV clearance and escape from immune surveillance. Therefore, inherited genetic alterations of *microRNAs* may affect susceptibility to HPV-associated OSCC.

Previous studies have demonstrated that *hsa-146a* was over-expressed in SCCHN cell lines and tumor tissues and *hsa-mir-149* was down-regulated in squamous cell carcinoma of the tongue and hsa*-mir-146a* polymorphism may also affect the miRNA expression [21], [28], [29]. Recent studies have demonstrated that single nucleotide polymorphisms (SNPs) of *miR146* G>C, *miR196* C>T, and *miR499* T>C may affect cancer risk and prognosis [30]–[33]. In one of our previous case-control studies, we explored the association between the four common SNPs (*miR146* rs2910164, *miR149* rs2292832, *miR196* rs11614913, and *miR499* rs3746444) and risk of head and neck cancers, but no significant association between each SNP and cancer risk was found [33]. Given the roles of *microRNAs* in regulating the immune and inflammatory response systems and thereby mediating HPV infection, we hypothesized that genetic variants in these four microRNAs alter the association between HPV16 seropositivity and risk of OSCC. To test this hypothesis, we evaluated the joint effect of HPV16 serology and these four common *microRNA* SNPs on risk of OSCC.

## Materials and Methods

### Study subjects

Patients with incident OSCC were recruited as part of an ongoing molecular epidemiology study of head and neck cancers through the Head and Neck Center at The University of Texas MD Anderson Cancer Center between December 1996 and October 2002. All patients were recruited without restrictions on age, sex, cancer stage, or histology and had newly diagnosed, histopathologically confirmed, untreated OSCC. Excluded from participation were patients with second primary tumors; primary tumors of the sinonasal tract, nasopharynx, hypopharynx, or larynx; primary tumors outside the upper aerodigestive tract; cervical metastases of unknown origin; and histopathologic diagnoses other than SCC. In addition, patients who had received a blood transfusion in the past 6 months or who were receiving immunosuppressive therapy were excluded. Among patients initially contacted for participation, approximately 95% of eligible incident cases agreed to participate in the study.

A pool of cancer-free subjects was recruited from the Kelsey-Seybold Foundation, a multispecialty physician practice with multiple clinics throughout the Houston metropolitan area, and from healthy visitors who accompanied cancer patients to outpatient clinics at MD Anderson Cancer Center but were genetically unrelated to the cases. In this pool of cancer-free controls, each individual was first surveyed by means of a short questionnaire to determine his or her willingness to participate in research studies. Each eligible subject was subsequently interviewed to collect demographic and epidemiologic information, such as age, sex, ethnicity, smoking status, and alcohol consumption status. The overall response rate for the controls was approximately 78%. Excluded from participation were individuals with history of any cancer, history of immunosuppressive therapy, or blood transfusion in the past 6 months.

Smoking status was categorized as “ever smokers” (those who had smoked more than 100 cigarettes in their lifetime) and “never smokers” (those who had smoked equal to or fewer than 100 cigarettes in their lifetime). Drinking status was categorized as “ever drinkers” (those who had drunk alcoholic beverages at least once a week for more than 1 year) and “never drinkers” (those who never had such a pattern of drinking). Both Institutional Review Boards of UT MD Anderson Cancer Center and Kelsey-seybold approved the study, and written informed consent was obtained from all study subjects. In this study, 335 cancer-free controls were selected from the pool of potential controls by frequency matching with the patients by age (±5 years), sex, and smoking and drinking status. To avoid confounding factors due to ethnic characteristics, all study subjects included were non-Hispanic whites, who accounted for the vast majority of the cases.

### HPV16 serologic detection

HPV16 L1 virus-like particles generated from recombinant baculovirus-infected insect cells were used to test for antibody against HPV16 L1 capsid protein in the plasma of study participants by using a standard enzyme-linked immunosorbent assay, as described previously [34]–[36]. About 10% of the samples were randomly chosen for the repeated assay, and the results were 100% concordant with results of initial testing.

### *MicroRNA* genotyping

From each blood sample, a leukocyte pellet was obtained from the buffy coat by centrifugation of 3 mL of whole blood. The pellet was used for genomic DNA extraction with the DNA Blood Mini Kit (Qiagen Inc., Valencia, CA) according to the manufacturer's instructions. A polymerase chain reaction-restriction fragment-length polymorphism assay was used to amplify the fragments that contained SNPs of *miR146* rs2910164, *miR149* rs2292832, *miR196* rs11614913, and *miR499* rs3746444. The *microRNA* SNPs, primers, and restriction enzymes (New England Biolabs, Inc., Beverly, MA) used for each SNP are shown in **Table 1**. The genotyping assays were repeated for a randomly selected subgroup of about 10% of the samples, and the results of retesting were 100% concordant with results of initial testing.

**Table 1:** Genotyping Assays of *MicroRNA* SNPs.

*MicroRNA*	SNP (Base Change)	Primers Used (Sense/Antisense)	PCR Product	Enzyme	Digested Products
*miR146* rs2910164	G>C	5′-AGGAGGGGTCTTTGC ACCATC-3′ 5′-CCCAGCTGA AGA ACTGAA CTGCA -3′	113 bp	*Pst*I	G allele: 113 bp C allele: 89 bp/24 bp
*miR149* rs2292832	C>T	5′-ATGTCCAGGACCACA ACCTGT-3′ 5′-CACCTCTCACACCCCCTCAC-3′	337 bp	*Pvu*II	C allele: 337 bp T allele: 220 bp/117 bp
*miR196* rs11614913	C>T	5′-CAGCTGATCTGTGGCTTAGGT-3′ 5′-GAAAACCGACTGATGTAACCCAG-3	93 bp	*Mva*I	T allele: 93 bp C allele: 70 bp/23 bp
*miR499* rs3746444	C>T	5′-ACGGGAAGCAGCACAGACTTG-3′ 5′-TGTTTAACTCCTCTCCACGTGTAC-3′	52 bp	*Bsr*GI	C allele: 52 bp T allele: 27 bp/25 bp

### Statistical analysis

The distributions of selected demographic variables, tobacco smoking, alcohol consumption, *microRNA* genotypes, and HPV16 serology between cases and controls were evaluated using the chi-square test. To evaluate the association of HPV16 serologic status and *microRNA* genotypes with OSCC risk, odds ratios (ORs) and 95% confidence interval (CIs) were calculated using both univariate and multivariate logistic regression analyses. Logistic regression was also used to assess the potential interaction effects by evaluating departures from the models of additive and multiplicative interactions between selected variables. A more-than-additive interaction was suggested when OR_11_>OR_10_+OR_01_−1, for which OR_11_ = OR when both factors were present, OR_10_ = OR when only factor 1 was present and OR_01_ = OR when only factor 2 was present. A more-than-multiplicative interaction was suggested when OR_11_>OR_10_×OR_01_. We assessed the interaction by reporting the *P* values from the Wald test for testing the coefficients (β*_microRNA_*_polymorphism, HPV16 seropositivity_) were different from 0, where the interaction term consisted of the product of the two variables: *microRNA* polymorphism and HPV16 seropositivity. We also assessed the joint effects of HPV16 serology and *microRNA* genotypes on OSCC risk, and the joint effects were further stratified by tumor site and smoking status. Statistical Analysis System software (Version 9.2; SAS Institute, Cary, NC) was used for all statistical analyses. All tests were two-sided, and *P*<0.05 was considered the cutoff for statistical significance.

## Results

### Demographics and risk factors for study subjects

The final analysis included 325 OSCC cases and 335 controls. Of the 325 cases, 188 (57.8%) had SCCOP, and 137 (42.2%) had SCC of the oral cavity. The distributions of demographic characteristics and known OSCC risk factors are summarized in **Table 2**. As a result of frequency matching, there was no significant difference between patients and controls in distributions of age, sex, smoking status, and alcohol use (all *P*>0.05). However, we observed that HPV16 seropositivity was significantly more common in patients than in controls (*P*<0.001). HPV16 seropositivity was associated with risk for OSCC (OR, 3.2, 95% CI, 2.1–4.8), particularly for SCCOP (OR, 5.4, 95% CI, 3.7-8/9), but not for oral cavity cancers (OR, 0.8, 95% CI, 0.38–1.48), after adjusting for age, sex, smoking status, and drinking status.

**Table 2:** Distribution of Demographic and Risk Factors in OSCC Patients and Controls.

Characteristic			Cases (n = 325)					
	Controls* (n = 335)		Overall OSCC		SCCOP		SCC of oral cavity	
	No.	%	No.	%	No.	%	No.	%
Age, years								
<40	27	8.1	31	9.5	17	9.0	14	10.2
41–55	105	31.3	126	38.8	86	45.8	40	29.2
56–70	154	46.0	119	36.6	64	34.0	55	40.2
>70	49	14.6	49	15.1	21	11.2	28	20.4
Sex								
Male	269	80.3	241	74.1	155	82.4	86	62.8
Female	66	19.7	84	25.9	33	17.6	51	37.2
Tobacco smoking								
Ever	239	71.3	227	69.8	125	66.5	102	74.4
Never	96	28.7	98	30.2	63	33.5	35	25.6
Alcohol drinking								
Ever	240	71.6	250	76.9	150	79.8	100	73.0
Never	95	28.4	75	23.1	38	20.2	37	27.0
HPV16 serology^a^								
Positive	42	12.5	100	30.8	87	46.3	13	9.5
Negative	293	87.5	225	69.2	101	53.7	124	90.5

* Controls were selected to be frequency matched to cases on the factors shown in the table.

a There was a significant difference in HPV16 serology between the cases and controls.

### Association of *microRNA* variants with risk of OSCC

The genotype distributions of the four *microRNA* SNPs among the controls were all in agreement with the Hardy-Weinberg equilibrium (*P* = 0.535 for *miR146* rs2910164, *P* = 0.988 for *miR149* rs2292832, *P* = 0.783 for *miR196* rs11614913, and *P* = 0.489 for *miR499* rs3746444). Overall, we did not find any statistically significant association of any of the four *microRNA* SNPs with the risk of OSCC, but we did find that *miR146* rs2910164 and *miR499* rs3746444 had a minor effect on the risk of SCCOP (**Table 3**). After adjustment for age, sex, smoking status, alcohol use, and HPV16 serology, individuals with the combined *miR146* rs2910164 CG and CC genotypes had a higher risk of SCCOP than individuals with the GG genotype (adjusted OR, 1.9; 95% CI, 1.3–3.0), and individuals with the *miR499* rs3746444 combined CT and CC genotypes had a higher risk of SCCOP than individuals with the TT genotype (adjusted OR, 1.6; 95% CI, 1.0–2.4).

**Table 3:** Association of *MicroRNA* SNPs with OSCC Risk.

Genotype	Cases (n = 325)		Controls (n = 335)		*P* *	Adjusted OR (95% CI)†		
	No.	%	No.	%		Overall OSCC	SCCOP	SCC of Oral Cavity
*miR146* rs2910164					0.299			
GG (Ref.)	184	56.6	203	60.6		1.0	1.0	1.0
CG+CC	141	43.4	132	39.4		1.3 (0.9–1.8)	1.9 (1.3–3.0)	1.0 (0.6–1.4)
*miR149* rs2292832					0.947			
CC (Ref.)	158	48.6	162	48.4		1.0	1.0	1.0
CT+TT	167	51.4	173	51.6		0.9 (0.7–1.3)	1.0 (0.7–1.5)	0.9 (0.6–1.3)
*miR196* rs11614913					0.871			
CC (Ref.)	95	29.2	96	28.7		1.0	1.0	1.0
CT+TT	230	70.8	239	71.3		1.0 (0.7–1.4)	1.0 (0.7–1.6)	1.0 (0.6–1.5)
*miR499* rs3746444					0.060			
TT (Ref.)	184	56.6	214	63.9		1.0	1.0	1.0
CT+CC	141	43.4	121	36.1		1.3 (0.9–1.9)	1.6 (1.0–2.4)	1.1 (0.8–1.7)

* Genotype distributions for *miR146* rs2910164, *miR149* rs2292832, *miR196* rs11614913, and *miR499* rs3746444 between patients and controls.

† ORs were adjusted for age, sex, smoking, alcohol drinking, and HPV16 serology.

### Association of *microRNA* variants with risk of HPV16-associated OSCC

As shown in **Table 4**, the association between HPV16 serology and OSCC risk was modified by *microRNA* genetic variants. Specifically, compared with individuals with both *miR146* rs2910164 GG genotype and HPV16 seronegativity, those with both GG genotype and HPV16 seropositivity had an increased risk of OSCC (OR, 3.0; 95% CI, 1.8–5.0), and the risk was even higher among those with both CG or CC genotype and HPV16 seropositivity (OR, 4.7; 95% CI, 2.3–9.4). In contrast, compared with individuals with both *miR149* rs2292832 CT or TT genotype and HPV16 seronegativity, those with both CT or TT genotype and HPV16 seropositivity had an increased risk of OSCC (OR, 2.9; 95% CI, 1.7–5.0), and the risk was even higher among those with both CC genotype and HPV16 seropositivity (OR, 3.6; 95% CI, 1.9–6.6). Similar results were observed for the associations between *miR196* rs11614913 and *miR499* rs3746444 SNPs and risk of HPV16-associated OSCC (**Table 4**). Considering that the difference in the tumor HPV status between patients with SCCOP and oral cavity cancers might be attributed to different etiologies at the two different anatomic sites, we further investigated the modifying effect of each SNP on the association between HPV16 seropositivity and risk of SCCOP and oral cavity cancers (**Table 4**). We found that the modifying effects of *microRNA* variants on the risk associated with HPV16 seropositivity were pronounced for SCCOP but not for SCC of the oral cavity. Moreover, the modification effect may suggest an additive interaction. However, when we further performed tests for interaction between HPV16 seropositivity and *microRNA* variants for risk of OSCC as shown in **Table 5**, we found that the interaction between HPV16 seropositivity and each of these *microRNA* variants on the risk of OSCC was not statistically significant (all P values>0.05).

**Table 4:** Joint Effect of HPV16 Seropositivity and *MicroRNA* Genotypes on OSCC Risk.

HPV16 Status	Genotype	Cases (n = 325)		Controls (n = 335)		Adjusted OR (95% CI)*		
		No.	%	No.	%	Overall OSCC	SCCOP	SCC of Oral Cavity
	*miR146* rs2910164							
−	GG (Ref.)	120	36.9	173	51.6	1.0	1.0	1.0
−	CG+CC	105	32.3	120	35.8	1.2 (0.9–1.8)	1.0 (0.5–1.3)	1.7 (1.1–2.7)
+	GG	64	19.7	30	9.0	3.0 (1.8–5.0)	5.0 (2.9–5.0)	0.5 (0.2–1.4)
+	CG+CC	36	11.1	12	3.6	4.7 (2.3–9.4)	6.3 (2.9–13.3)	2.4 (0.9–6.4)
	*miR149* rs2292832							
**−**	CT+TT (Ref.)	114	35.1	148	44.2	1.0	1.0	1.0
**−**	CC	111	34.1	145	43.3	1.0 (0.7–1.4)	1.0 (0.6–1.5)	1.1 (0.7–1.6)
+	CT+TT	53	16.3	25	7.4	2.9 (1.7–5.0)	5.2 (2.9–9.4)	0.6 (0.2–1.6)
+	CC	47	14.5	17	5.1	3.6 (1.9–6.6)	6.0 (3.1–11.6)	1.0 (0.4–2.5)
	*miR196* rs11614913							
−	CC (Ref.)	64	19.7	82	24.5	1.0	1.0	1.0
−	CT+TT	161	49.5	211	63.0	1.0 (0.6–1.4)	1.0 (0.6–1.7)	0.9 (0.6–1.4)
+	CC	31	9.6	14	4.2	2.8 (1.4–5.8)	5.7 (2.6–12.5)	0.5 (0.1–1.8)
+	CT+TT	69	21.2	28	8.3	3.2 (1.8–5.6)	6.0 (3.1–11.3)	0.8 (0.4–1.9)
	*miR499* rs3746444							
**−**	TT (Ref.)	124	38.1	186	55.5	1.0	1.0	1.0
**−**	CT+CC	101	31.1	107	31.9	1.4 (1.0–2.0)	1.1 (0.7–1.8)	1.6 (1.0–2.5)
+	TT	60	18.5	28	8.4	3.4 (2.0–5.6)	5.5 (3.2–9.5)	0.9 (0.4–2.2)
+	CT+CC	40	12.3	14	4.2	4.1 (2.1–8.0)	6.9 (3.4–13.7)	1.0 (0.3–2.8)

* ORs were adjusted for age, sex, smoking, and alcohol drinking.

**Table 5:** Modifying effect of *microRNA* variants on association between HPV16 Seropositivity and OSCC risk.

Genotype	HPV16+(n = 142)		HPV16−(n = 518)		Adjusted OR (95% CI)†	P_int._
	Case	Control	Case	Control		
*miR146* rs2910164						0.633
GG (Ref.)	64	30	120	173	3.0 (1.8–5.0)	
CG+CC	36	12	105	120	3.9 (1.9–8.2)	
*miR149* rs2292832						0.700
CC (Ref.)	47	17	111	145	2.9 (1.4–6.1)	
CT+TT	53	25	114	148	3.3 (2.0–5.4)	
*miR196* rs11614913						0.627
CC (Ref.)	31	14	64	82	3.7 (2.0–6.8)	
CT+TT	69	28	161	211	3.2 (1.8–5.6)	
*miR499* rs3746444						0.761
TT (Ref.)	60	28	124	186	3.3 (2.0–5.6)	
CT+CC	40	14	101	107	2.8 (1.4–5.6)	

† ORs were adjusted for age, sex, smoking, and alcohol drinking.

P_int_.:P values for interaction.

### Stratified analyses of joint effects of HPV seropositivity and *microRNA* variants on OSCC risk by smoking status

In order to explore whether the effects of *microRNA* SNPs on the risk of HPV16-associated OSCC were confounded by smoking status, we performed stratified analyses of joint effects of HPV16 seropositivity and *microRNA* variants on risk of OSCC by smoking status (**Table 6**). Overall, we observed that for each SNP, the risk of HPV16-associated OSCC was much stronger in never smokers than in ever smokers and such effect modification was particularly pronounced in SCCOP.

**Table 6:** Joint Effect of HPV16 Seropositivity and *MicroRNA* Genotypes on OSCC Risk Stratified by Smoking Status.

HPV16 Status	*miRNA genotypes*	Never Smokers	Ever Smokers	Adjusted OR, (95% CI)* Overall OSCC		Adjusted OR, (95% CI)* SCCOP	
		Cases/Controls	Cases/Controls	Never Smokers	Ever Smokers	Never Smokers	Ever Smokers
	*miR146* rs2910164	n = 98/96	n = 227/239				
−	GG(Ref.)	32/48	88/125	1.0	1.0	1.0	1.0
−	CG+CC	27/40	78/80	1.0 (0.5–1.9)	1.3 (0.9–2.0)	1.0(0.2–1.3)	1.1(0.6–1.9)
+	GG	28/6	36/24	8.5 (3.1–23.7)	2.0 (1.1–3.7)	12.3(4.1–37.0)	3.9(2.1–7.4)
+	CG+CC	11/2	25/10	11.5 (2.3–57.3)	4.0 (1.8–9.0)	17.0(3.2–88.5)	5.0(2.1–11.9)
	*miR149* rs2292832	(n = 98/96)	(n = 227/239)				
−	CT+TT(Ref.)	35/36	79/112	1.0	1.0	1.0	1.0
−	CC	24/52	87/93	0.5 (0.3–1.1)	1.4 (0.9–2.1)	1.0(0.1–10.0)	1.4(0.8–2.5)
+	CT+TT	18/6	35/19	4.2 (1.4–12.5)	2.8 (1.5–5.3)	5.7(1.7–18.9)	5.6(2.8–11.1)
+	CC	21/2	26/15	14.0 (3.0–66.8)	2.5 (1.2–5.1)	24.5(4.8–124.5)	4.0(1.8–8.8)
	*miR196* rs11614913	(n = 98/96)	(n = 227/239)				
−	CC(Ref.)	13/25	51/57	1.0	1.0	1.0	1.0
−	CT+TT	46/63	115/148	1.5 (0.6–3.3)	0.8 (0.5–1.3)	3.1(0.8–12.0)	0.8(0.5–1.5)
+	CC	9/4	22/10	5.7 (1.4–23.7)	2.5 (1.1–5.8)	20.4(3.4–121.3)	4.6(1.8–11.6)
+	CT+TT	30/4	39/24	19.5 (5.3–71.2)	1.8 (1.0–3.5)	73.8(13.5–402.0)	3.1(1.5–6.4)
	*miR499* rs3746444	(n = 98/96)	(n = 227/239)				
−	TT (Ref.)	34/61	90/125	1.0	1.0	1.0	1.0
−	CT+CC	25/27	76/80	1.6 (0.8–3.2)	1.3 (0.8–1.9)	1.1(0.4–2.8)	1.1(0.6–1.9)
+	TT	23/6	37/22	9.4 (3.4–26.4)	2.5 (1.3–4.5)	16.8(5.4–51.8)	3.8(2.0–7.4)
+	CT+CC	16/2	24/12	14.9 (3.1–70.9)	2.6 (1.2–5.5)	22.1(4.4–112.0)	5.0(2.2–11.1)

* ORs were adjusted for age, sex, and alcohol drinking.

## Discussion

In this study, overall, we did not observe a significant main effect of each SNP of these *microRNAs* on risk of OSCC, but we did observe a significant association between *miR146* rs2910164 and *miR*499 rs3746444 and a moderately increased risk of SCCOP. However, we found that the joint effect of HPV16 seropositivity and each of these *microRNA* SNPs increased the risk of OSCC, although we did not observe any significant interaction for such joint effect on risk of OSCC. Moreover, such effect modification was more pronounced for SCCOP as opposed to SCC of oral cavity and in never smokers than in ever smokers. Our results are in agreement with the characteristics of SCCOP associated with HPV infection, indicating that *microRNA* SNPs might play a role in the development of HPV16-associated SCCOP.

*MicroRNAs* act as omnipresent regulators of gene expression and are involved in many cellular processes, including inflammation and immune responses. Recent studies have demonstrated that *microRNAs* participate in mediating inflammatory and cytokine signaling as well as innate and acquired immune response to viral infection by targeting critical elements in inflammatory signaling pathways [37], [38]. *MicroRNAs* also modify viral-host interactions, which play central roles in the development and progression of infection-associated tumors.

Therefore, functional genetic polymorphisms of *microRNAs* may lead to individual variations in immune function, inflammation, and apoptosis that modify viral immune escape, antiviral defense, and evasion of apoptosis, leading to modification of the risk of infection-associated cancers. Numerous studies have shown that some *microRNAs* are up-regulated, while other *microRNAs* are down-regulated in head and neck cancers [39]–[41]. Such increased or decreased expression of *microRNAs* may be associated with the development, progression, and prognosis of head and neck cancers [24], [42].

Recently, several authors have reported associations between *microRNA SNPs* and the risk of head and neck cancers. One study indicated that the *miR196* rs11614913 variant reduced the risk of head and neck cancers [32]. Another study indicated that *miR146* rs2910164, *miR149* rs2292832, and *miR196* rs11614913 did not modify the risk of head and neck cancers independently of HPV infection but that *miR499* rs3746444 moderately reduced the risk of head and neck cancers [33]. The conflicting results may be attributed to many factors, such as different anatomical tumor sites, lack of information about other confounders, and small sample sizes. For example, the previous studies, although larger than our study, had mixed tumor sites without stratification by HPV infection status, whereas in our current study, we assessed the joint effects of *microRNA SNPs* and HPV16 infection on OSCC risk. HPV16 infection has recently been identified as one of the primary etiologic factors for causes of SCCOP, but oral cavity and laryngeal cancer mainly result from tobacco and alcohol consumption. In our current study, although we had a smaller study size, we found that *microRNA* SNPs and HPV16 seropositivity may function jointly in the development of OSCC, particularly in patients with SCCOP and in never smokers.

Although the precise mechanism by which the common *microRNA* genetic variants and HPV16 infection jointly play a role in the development of OSCC has not been fully clarified, a joint effect of *microRNAs* and HPV16 infection on susceptibility to OSCC is biologically plausible. HPV16 is oncogenic: it encodes viral oncoproteins E6 and E7, which inhibit p53 and Rb cell cycle tumor suppressors. *MicroRNA* variants might functionally affect expression of genes involved in myriad cellular processes, including inflammation and immune response pathways, subsequently controlling the host's ability to clear HPV and HPV's ability to escape immune surveillance [37]. Therefore, our data suggest that HPV and *microRNA* variants might act jointly in the development of OSCC. However, further studies are required to validate the hypothesized functionality of these SNPs.

In the present study, our analysis stratified by tumor site showed that the effect modification was more pronounced for SCCOP than for oral cavity SCC, reinforcing the concept that there are differences in the etiology of cancers of the oropharynx and oral cavity. Additionally, the analysis further stratified by smoking status for each SNP showed that the joint effect of *microRNA* SNPs and HPV seropositivity on the risk of OSCC was much stronger in never smokers than in ever smokers. These data further support the notion that risk genotypes of the four common SNPs of *microRNAs* may be involved in the development of HPV-associated OSCC in non-Hispanic white never smokers. However, the modifying effect of each of *microRNA* SNPs on risk of OSCC associated with HPV16 was not statistically significant. This lack of significance could be either because there was no such interaction effect in these subgroups or because the small sample sizes in each substratum limited the statistical power to detect a significant interaction effect. Therefore, the significance and degree of such interaction in each subgroup needs to be further investigated in future studies with larger sample sizes.

Although the present study minimized potential confounding factors, several limitations should be considered. First, a possible selection bias in patient recruitment cannot be ruled out as our study was a hospital-based case-control study and as the cases and controls were not selected from the same target population. In addition, only non-Hispanic white subjects were included in our study. It is therefore uncertain whether these results are generalizable to other ethnic populations. Second, the effects of potential demographic confounding factors were minimized by adequate frequency matching on age, sex, smoking status, and alcohol use, but stratified analyses included a limited number of individuals in each subgroup. As such, the role of these SNPs and HPV16 in OSCC will require confirmation in larger studies in different populations. Third, because individual patients may have had differences in their immune response to HPV16 infection or antibody instability, HPV16 seropositivity may not reflect the tumor's true HPV16 status, leading to possible misclassifications of some patients as HPV16 seronegative who had tumors that actually were HPV16 DNA positive. This misclassification could result in a major selection bias for the estimates of association. Finally, lack of information on changes in sexual practices in current study did not allow us to evaluate its potential influence on risk of HPV-associated OSCC, particularly SCCOP. Therefore, our future studies should include the patients with tumor HPV status and sexual behaviors to further evaluate our findings once such information become available. Nevertheless, the use of serologic status may provide a possibility to include a cancer-free control group for the present case-control study design.

In conclusion, our results indicate that *microRNA* variants may modify the risk of HPV16-associated OSCC, particularly in patients with SCCOP and in never smokers. However, further prospective studies with larger sample sizes are needed to validate our findings.

## References

[1] Gillison ML (2007) Current topics in the epidemiology of oral cavity and oropharyngeal cancers. Head Neck 29: 779–792. 17230556 10.1002/hed.20573

[2] Carvalho AL, Nishimoto IN, Califano JA, Kowalski LP (2005) Trends in incidence and prognosis for head and neck cancer in the United States: a site-specific analysis of the SEER database. Int J Cancer 114: 806–816. 15609302 10.1002/ijc.20740

[3] Siegel R, Naishadham D, Jemal A (2012) Cancer statistics, 2012. CA Cancer J Clin 62: 10–29. 22237781 10.3322/caac.20138

[4] Vokes EE, Weichselbaum RR, Lippman SM, Hong WK (1993) Head and neck cancer. N Engl J Med 328: 184–194. 8417385 10.1056/NEJM199301213280306

[5] Sturgis EM, Cinciripini PM (2007) Trends in head and neck cancer incidence in relation to smoking prevalence: an emerging epidemic of human papillomavirus-associated cancers? Cancer 110: 1429–1435. 17724670 10.1002/cncr.22963

[6] Chaturvedi AK, Engels EA, Anderson WF, Gillison ML (2008) Incidence trends for human papillomavirus-related and -unrelated oral squamous cell carcinomas in the United States. J Clin Oncol 26: 612–619. 18235120 10.1200/JCO.2007.14.1713

[7] Ernster JA, Sciotto CG, O'Brien MM, Finch JL, Robinson LJ, et al (2007) Rising incidence of oropharyngeal cancer and the role of oncogenic human papilloma virus. Laryngoscope 117: 2115–2128. 17891052 10.1097/MLG.0b013e31813e5fbb

[8] Herrero R, Castellsagué X, Pawlita M, Lissowska J, Kee F, et al (2003) Human papillomavirus and oral cancer: the International Agency for Research on Cancer multicenter study. J Natl Cancer Inst 95: 1772–1783. 14652239 10.1093/jnci/djg107

[9] Fakhry C, Gillison ML (2006) Clinical implications of human papillomavirus in head and neck cancers. J Clin Oncol 24: 2606–2611. 16763272 10.1200/JCO.2006.06.1291 PMC4696042

[10] Gillison ML, Koch WM, Capone RB, Spafford M, Westra WH, et al (2000) Evidence for a causal association between human papillomavirus and a subset of head and neck cancers. J Natl Cancer Inst 92: 709–720. 10793107 10.1093/jnci/92.9.709

[11] Bartel DP (2004) MicroRNAs: genomics, biogenesis, mechanism, and function. Cell 116: 281–297. 14744438 10.1016/s0092-8674(04)00045-5

[12] Bentwich I, Avniel A, Karov Y, Aharonov R, Gilad S, et al (2005) Identification of hundreds of conserved and nonconserved human microRNAs. Nat. Genet 37: 766–770. 10.1038/ng1590 15965474

[13] Griffiths-Jones S, Grocock RJ, van Dongen S, Bateman A, Enright AJ (2006) miRBase: microRNA sequences, targets and gene nomenclature. Nucleic Acids Res 34 (Database issue) D140–144. 16381832 10.1093/nar/gkj112 PMC1347474

[14] Johnson SM, Grosshans H, Shingara J, Byrom M, Jarvis R, et al (2005) RAS is regulated by the let-7 microRNA family. Cell 120: 635–647. 15766527 10.1016/j.cell.2005.01.014

[15] Kloosterman WP, Plasterk RH (2006) The diverse functions of microRNAs in animal development and disease. Dev Cell 11: 441–450. 17011485 10.1016/j.devcel.2006.09.009

[16] Stefani G, Slack FJ (2008) Small non-coding RNAs in animal development. Nat Rev Mol Cell Biol 9: 219–230. 18270516 10.1038/nrm2347

[17] Esquela-Kerscher A, Slack FJ (2006) Oncomirs - microRNAs with a role in cancer. Nat Rev Cancer 6: 259–269. 16557279 10.1038/nrc1840

[18] Osada H, Takahashi T (2007) MicroRNAs in biological processes and carcinogenesis. Carcinogenesis 28: 2–12. 17028302 10.1093/carcin/bgl185

[19] Lu J, Getz G, Miska EA, Alvarez-Saavedra E, Lamb J, et al (2005) MicroRNA expression profiles classify human cancers. Nature 435: 834–838. 15944708 10.1038/nature03702

[20] Yanaihara N, Caplen N, Bowman E, Seike M, Kumamoto K, et al (2006) Unique microRNA molecular profiles in lung cancer diagnosis and prognosis. Cancer Cell 9: 189–198. 16530703 10.1016/j.ccr.2006.01.025

[21] Kozaki K, Imoto I, Mogi S, Omura K, Inazawa J (2008) Exploration of tumor-suppressive microRNAs silenced by DNA hypermethylation in oral cancer. Cancer Res 68: 2094–2105. 18381414 10.1158/0008-5472.CAN-07-5194

[22] Tran N, McLean T, Zhang X, Zhao CJ, Thomson JM, et al (2007) MicroRNA expression profiles in head and neck cancer cell lines. Biochem Biophys Res Commun 358: 12–17. 17475218 10.1016/j.bbrc.2007.03.201

[23] Bloomston M, Frankel WL, Petrocca F, Volinia S, Alder H, et al (2007) MicroRNA expression patterns to differentiate pancreatic adenocarcinoma from normal pancreas and chronic pancreatitis. JAMA 297: 1901–1908. 17473300 10.1001/jama.297.17.1901

[24] Chang SS, Jiang WW, Smith I, Poeta LM, Begum S, et al (2008) MicroRNA alterations in head and neck squamous cell carcinoma. Int J Cancer 123: 2791–2797. 18798260 10.1002/ijc.23831 PMC3184846

[25] Iorio MV, Ferracin M, Liu CG, Veronese A, Spizzo R, et al (2005) MicroRNA gene expression deregulation in human breast cancer. Cancer Res 65: 7065–7070. 16103053 10.1158/0008-5472.CAN-05-1783

[26] Iorio MV, Ferracin M, Liu CG, Veronese A, Spizzo R, et al (2008) MicroRNAs and the immune response. Trends Immunol 29: 343–351. 18515182 10.1016/j.it.2008.04.004

[27] Pedersen I, David M (2008) MicroRNAs in the immune response. Cytokine 43: 391–394. 18701320 10.1016/j.cyto.2008.07.016 PMC3642994

[28] Jazdzewski K, Murray EL, Franssila K, Jarzab B, Schoenberg DR, et al (2008) Common SNP in pre-miR-146a decreases mature miR expression and predisposes to papillary thyroid carcinoma. Proc Natl Acad Sci U S A 105: 7269–7274. 18474871 10.1073/pnas.0802682105 PMC2438239

[29] Wong TS, Liu XB, Wong BY, Ng RW, Yuen AP, et al (2008) Mature miR-184 as Potential Oncogenic microRNA of Squamous Cell Carcinoma of Tongue. Clin Cancer Res 14: 2588–2592. 18451220 10.1158/1078-0432.CCR-07-0666

[30] Hu Z, Chen J, Tian T, Zhou X, Gu H, et al (2008) Genetic variants of miRNA sequences and non-small cell lung cancer survival. J Clin Invest 118: 2600–2608. 18521189 10.1172/JCI34934 PMC2402113

[31] Hu Z, Liang J, Wang Z, Tian T, Zhou X, et al (2009) Common genetic variants in pre-microRNAs were associated with increased risk of breast cancer in Chinese women. Hum Mutat 30: 79–84. 18634034 10.1002/humu.20837

[32] Christensen BC, Avissar-Whiting M, Ouellet LG, Butler RA, Nelson HH, et al (2010) Mature microRNA sequence polymorphism in MIR196A2 is associated with risk and prognosis of head and neck cancer. Clin Cancer Res 16: 3713–3720. 20501619 10.1158/1078-0432.CCR-10-0657 PMC2914465

[33] Liu Z, Li G, Wei S, Niu J, El-Naggar AK, et al (2010) Genetic variants in selected pre-microRNA genes and the risk of squamous cell carcinoma of the head and neck. Cancer 116: 4753–4760. 20549817 10.1002/cncr.25323 PMC3030480

[34] Kirnbauer R, Hubbert NL, Wheeler CM, Becker TM, Lowy DR, et al (1994) A virus-like particle enzyme-linked immunosorbent assay detects serum antibodies in a majority of women infected with human papillomavirus type 16. J Natl Cancer I 86: 494–499. 10.1093/jnci/86.7.494 PMC3935441 8133532

[35] Dahlstrom KR, Adler-Storthz K, Etzel CJ, Liu Z, Dillon L, et al (2003) Human papillomavirus type 16 infection and squamous cell carcinoma of the head and neck in never-smokers: a matched pair analysis. Clin Cancer Res 9: 2620–2626. 12855639

[36] Chen X, Sturgis EM, Lei D, Dahlstrom K, Wei Q, et al (2010) Human papillomavirus seropositivity synergizes with MDM2 variants to increase the risk of oral squamous cell carcinoma. Cancer Res 70: 7199–7208. 20736372 10.1158/0008-5472.CAN-09-4733 PMC2940953

[37] Sonkoly E, Ståhle M, Pivarcsi A (2008) MicroRNAs and immunity: novel players in the regulation of normal immune function and inflammation. Semin Cancer Biol 18: 131–140. 18291670 10.1016/j.semcancer.2008.01.005

[38] Sonkoly E, Wei T, Janson PC, Sääf A, Lundeberg L, et al (2007) MicroRNAs: novel regulators involved in the pathogenesis of psoriasis? PLoS One 2: e610. 17622355 10.1371/journal.pone.0000610 PMC1905940

[39] Hebert C, Norris K, Scheper MA, Nikitakis N, Sauk JJ (2007) High mobility group A2 is a target for miRNA-98 in head and neck squamous cell carcinoma. Mol Cancer 6: 5. 17222355 10.1186/1476-4598-6-5 PMC1783857

[40] Liu X, Chen Z, Yu J, Xia J, Zhou X (2009) MicroRNA profiling and head and neck cancer. Comp Funct Genomics 837514. 19753298 10.1155/2009/837514 PMC2688814

[41] Guo Y, Fu W, Chen H, Shang C, Zhong M (2012) MiR-24 functions as a tumor suppressor in Hep2 laryngeal carcinoma cells partly through down-regulation of the S100A8 protein. Oncol Rep 27: 1097–2103. 22139384 10.3892/or.2011.1571 PMC3583566

[42] Henson BJ, Bhattacharjee S, O'Dee DM, Feingold E, Gollin SM (2009) Decreased expression of miR-125b and miR-100 in oral cancer cells contributes to malignancy. Genes Chromosomes Cancer 48: 569–582. 19396866 10.1002/gcc.20666 PMC2726991

